# gndDb, a Database of Partial *gnd* Sequences To Assist with Analysis of Escherichia coli Communities Using High-Throughput Sequencing

**DOI:** 10.1128/MRA.00476-19

**Published:** 2019-08-15

**Authors:** Adrian L. Cookson, David W. Lacher, Flemming Scheutz, David A. Wilkinson, Patrick J. Biggs, Jonathan C. Marshall, Gale Brightwell

**Affiliations:** a AgResearch Limited, Hopkirk Research Institute, Palmerston North, New Zealand; b mEpiLab, Hopkirk Research Institute, School of Veterinary Science, Massey University, Palmerston North, New Zealand; c Division of Molecular Biology, Office of Applied Research and Safety Assessment, Center for Food Safety and Applied Nutrition, U.S. Food and Drug Administration, Laurel, Maryland, USA; d Department of Microbiology and Infection Control, Statens Serum Institut, Copenhagen, Denmark; e School of Fundamental Sciences, Massey University, Palmerston North, New Zealand; f New Zealand Food Science and Safety Research Centre, Massey University, Palmerston North, New Zealand; Broad Institute

## Abstract

The use of culture methods to detect Escherichia coli diversity does not provide sufficient resolution to identify strains present at low levels. Here, we target the hypervariable *gnd* gene and describe a database containing 534 distinct partial *gnd* sequences and associated O groups for use with culture-independent E. coli community analysis.

## ANNOUNCEMENT

Current culture-dependent studies to investigate Escherichia coli diversity in fecal and environmental samples often fail to identify strains that are present in low numbers. Our previous work using sequencing of metabarcoded amplicons has targeted the hypervariable *gnd* gene to provide a comprehensive analysis of E. coli community structure from complex samples such as feces (1). The *gnd* gene encodes 6-phosphogluconate dehydrogenase, the third enzyme in the pentose phosphate pathway, and is found in most *Enterobacteriaceae* (2–4). Usually, *gnd* is found adjacent to the highly recombinatorial O-antigen biosynthesis gene cluster (O-AGC) (5, 6), a region of the E. coli chromosome prone to horizontal gene transfer and recombination (7), which influences the O-group structure and cell surface antigenicity as the outermost component of the lipopolysaccharide (LPS) moiety. Although having no role in O-antigen biosynthesis, *gnd* has been described as a passive hitchhiker of recombination events influencing LPS antigenic changes (4).

By targeting *gnd* polymorphisms and adopting culture-independent methods, our previous work provided an indication of intestinal E. coli diversity and in parallel developed a *gnd* database for cross-referencing purposes using O-AGC DNA sequence data from distinct O groups (1). However, the increasing availability and analysis of whole-genome sequencing (WGS) data from E. coli (and Shigella) isolates have provided new insights as to the range of different E. coli O groups according to incongruent *wzx* and *wzy* (provisional OXY designations) or *wzm* and *wzt* (provisional OMT designations) gene sequences and the identification of six novel O groups (8). By isolating and examining the *gnd* gene from E. coli and *Shigella* draft genome assemblies described in recent studies (9, 10) and in other studies where novel O-AGCs have been submitted to GenBank (5, 6, 8), we have identified novel *gnd* alleles that have been included in a database resource that may be used for analysis of E. coli communities using amplicon sequencing. Analysis of WGS data from E. coli (and *Shigella*) with novel O groups has provided evidence for the identification of 534 distinct *gnd* sequence types (gSTs), each 284 bp in length, forming the core of this database.

The full-length *gnd* gene of 1,407 bp precludes its use in its entirety as a tool for culture-independent amplicon sequencing studies using the Illumina platform; therefore, the 284-bp region spanning nucleotide positions 443 to 727 is targeted for use in E. coli community analysis studies. The hypervariable nature of *gnd* also restricts suitable PCR primer sites for the generation of amplicons of a suitable size for routine high-throughput sequencing using the Illumina platform. This *gnd* database may also provide some assistance in the identification of novel O groups and offer a resource for E. coli subtyping using conventional dideoxy Sanger sequencing methods as a primary screen for subsequent WGS analysis (11).

### Data availability.

The DNA sequences of the 534 distinct gSTs are available in FASTA format from GitHub (https://github.com/mEpiLab/gnd) and are accompanied by a spreadsheet which provides matching O groups for each gST and a representative accession number of matching draft genome assemblies or submitted nucleotide sequences. The database and list of matching O groups will be curated and updated as further WGS data are made available.
